# Diagnosis and treatment of phrenic nerve hourglass constriction in patients with Parsonage-Turner syndrome

**DOI:** 10.3171/2022.10.FOCVID22105

**Published:** 2023-01-01

**Authors:** Travis S. CreveCoeur, Christopher J. Visco, Mark E. Ginsburg, Christopher J. Winfree

**Affiliations:** 1 Department of Neurological Surgery, Columbia University Irving Medical Center, New York;; 2 Department of Rehabilitation and Regenerative Medicine, Columbia University College of Physicians and Surgeons, New York;; 3 Department of Thoracic Surgery, Columbia University Irving Medical Center, New York, New York

**Keywords:** phrenic nerve, Parsonage-Turner syndrome, diaphragm paralysis

## Abstract

Phrenic nerve injury can occur anywhere along its course and clinically results in diaphragm paralysis. Although most patients with Parsonage-Turner syndrome and phrenic nerve dysfunction improve without treatment, some patients do not recover spontaneously. In these cases, an initial autoimmune response produces scarring along the affected nerve(s). This scar, known as an hourglass constriction, causes focal compression of the nerve at the site of the scar, which prevents the nerve from spontaneously recovering. Thus, the authors present a unique case of phrenic nerve injury secondary to Parsonage-Turner syndrome that improved with internal neurolysis.

The video can be found here: https://stream.cadmore.media/r10.3171/2022.10.FOCVID22105

## Transcript

This is Travis CreveCoeur from the Department of Neurological Surgery at Columbia University Medical Center.

### 0:27 Case and Initial Workup.

This case is a 64-year-old male with a remote history of a fall who is neurologically intact. Two weeks later, he developed exertional shortness of breath.

Initial workup demonstrated a chest x-ray which showed an elevated right hemidiaphragm. A right phrenic nerve pathology was suspected.

He was referred to our diaphragm paralysis center, and a right phrenic neuropathy was confirmed with SNIFF and pulmonary functional tests.

### 0:55 Diagnosis.

In review, we have a healthy male with a minor fall with no evidence of acute nerve injury initially, but he presented with a delayed diaphragmatic paralysis. There is no obvious injury mechanism evident. This pattern is consistent with a painless variant of Parsonage-Turner syndrome.

### 1:12 Diagnosis of Parsonage-Turner Syndrome.

Parsonage-Turner syndrome is a disorder where the body’s immune system produces antibodies that attack the peripheral nerves, with the phrenic nerve involved in about 7% of these cases.^1^ The immune response can be triggered by stress of any kind such as viral illness, vaccination, surgery, or biomechanical forces. In this case, biomechanical forces played a role in triggering Parsonage-Turner syndrome, as a nerve can be subjected to a focal stretch or a compression that initiates this immune response.

Patients often experience a prodrome of neck, arm, and shoulder pain. Once the prodrome resolves, the patients become aware of neurological deficits. It can be any pattern or combination of motor or sensory deficits. This pattern is typically patchy and often not consistent with an injury mechanism. Patients who have phrenic nerve involvement may experience shortness of breath with exertion, as seen with this case.

Most patients with Parsonage-Turner syndrome with phrenic nerve involvement improve with conservative treatment; however, some patients do not recover spontaneously. In these cases, the initial autoimmune response produces scarring along the affected nerves. This scar, called an hourglass constriction, is a well-described pathological finding in Parsonage-Turner syndrome and causes focal compression of the nerve. This is thought to be a reason why a subset of Parsonage-Turner patients do not make a full recovery.^2^ Given the patient’s clinical presentation and suspicion for Parsonage-Turner syndrome, we investigated the possibility that an hourglass constriction could be present. We therefore obtained high-resolution phrenic nerve imaging.

### 2:52 Imaging Confirmation.

The patient initially underwent MR neurography.^3^ The phrenic nerve was not well visualized due to motion artifact. We additionally obtained a high-resolution ultrasound which then showed the hourglass constriction. It was roughly 1 cm rostral to the rostral edge of the clavicle at the intersection of the phrenic nerve and a transverse cervical artery, all of which was overlying the nerve.

The results were documented real time, but the images were not saved.

### 3:15 Rationale for Surgery.

With diagnosis of Parsonage-Turner syndrome confirmed, the decision to surgically decompress the hourglass constriction was made, given the fact that we were still within 1 year from the symptoms to allow for reinnervation.^4^

Surgery was performed jointly with thoracic and neurosurgery.^5,6^

### 3:31 Positioning.

The patient is in the supine position and a variety of structures are marked. The "SCM" represents the posterior border of the sternocleidomastoid muscle. The "C" represents the rostral edge of the clavicle, the "SN" refers to the sternal notch, and the "A" is the acromion. The asterisk marks the location of the hourglass constriction.

We performed a standard supraclavicular exposure of the phrenic nerve as it runs across the anterior surface of the anterior scalene muscle. Once the nerve was exposed, we identified the hourglass constriction and the overlying vessel as described on the ultrasound. This vessel is on the left side of the screen. We’ve divided the vessel from the nerve to enhance our exposure.^7^

### 3:57 Internal Neurolysis.

Here noted is the fascicle involved in the hourglass constriction. We note that it is significantly larger above and below the hourglass constriction compared to adjacent uninvolved fascicles. This is a characteristic finding associated with hourglass constriction seen in Parsonage-Turner syndrome.

The next step at this point is to begin the internal neurolysis by incising the epineurium. This is done under high magnification with a combination of an 11 blade and straight microscissors. Intermittent stimulation was then performed up to 2.0 mA, however with no response seen. And you’ll note as we open the epineurium, the fascicle appears to be under pressure and begins to gently herniate out of our exposure, suggesting the fascicle is indeed under pressure.

The hourglass constriction is actually at the very center part of exposure, and we can’t easily see it just yet. But as we open the epineurium more extensively, the hourglass constriction comes into view. Here we’re exposing the affected fascicle and you can see the hourglass constriction right in the center of our exposure.

Our next maneuver is to incise the perineurium of the involved fascicle. This relieves the hourglass constriction and decompresses the involved fascicle. As we open up the perineum, the fascicle begins to herniate out, suggesting that it is in fact under pressure. Once the epineurium and perinium are fully incised across the constriction and enlarged fascicle, the decompression is complete.

Once done, the nerve plumps up, there’s no evidence of any constriction anymore, the fascicles are their own normal sizes and not under pressure. We apply a topical hemostatic agent so that we avoid cauterizing the nerve.

Please note that no further direct stimulation was performed as we visualized the nerve decompression. The wound is closed in the usual multilayered fashion. The patient is sent home after an appropriate period of observation as an outpatient.

### 6:05 Follow-Up.

The initial 2-week follow-up chest x-ray showed no change from baseline. The 6-month follow-up also showed no change from baseline. However, the 2-year follow-up chest x-ray showed resolution of the patient’s elevated hemidiaphragm. Additionally, the patient’s exertional dyspnea was resolved by the 2-year follow-up visit.

This is the first patient with Parsonage-Turner syndrome with hourglass constriction that we treated in our diaphragm paralysis center.

We look forward to reporting on additional patients as 2-year follow-up data becomes available.

### 6:36 Conclusion.

Phrenic neuropathy is a well-recognized presentation of Parsonage-Turner syndrome. Hourglass constrictions are a recognized complication of Parsonage-Turner syndrome. This represents the first patient with Parsonage-Turner syndrome with an hourglass constriction that has been surgically treated with internal neurolysis.

Thank you for watching the video.

### Disclosures

Dr. Visco: personal fees from AMSSM, outside the submitted work; and board member of the Association of Academic Physiatry.

### Author Contributions

Primary surgeon: Winfree. Assistant surgeon: CreveCoeur. Editing and drafting the video and abstract: CreveCoeur, Visco, Winfree. Critically revising the work: CreveCoeur, Visco, Winfree. Reviewed submitted version of the work: all authors. Approved the final version of the work on behalf of all authors: CreveCoeur. Supervision: Winfree.

## Supplemental Information

### Patient Informed Consent

The necessary patient informed consent was obtained in this study.
